# Address of Dr. J. G. Fredericks

**Published:** 1883-09

**Authors:** 


### THE DENTAL REGISTER.

VOL. XXXVII.] **SEPTEMBER, 1883.** [No. 9.

### COMMUNICATIONS.

### Address of Dr. J. G. Fredericks,

ACTING PRESIDENT OF THE AMERICAN DENTAL ASSOCIATION.

GENTLEMEN—By divine permission we are here convened, to confer upon the high interests of our profession; but the pleasure which I experience in again beholding so many familiar faces is mingled with emotions of sorrow, that from our midst are forever removed two of our best known and most valued members. Dr. Marshall H. Webb and our late President, Dr. Wm. H. Goddard, since our last reunion have both embarked upon the vast and mysterious ocean of eternity. As the successor of one, as the friend of both, and in my official capacity I feel it my first duty to voice the sentiment of all assembled here, and twine one single flower in the great wreath of respect, of love and regret already woven in their honor by the profession at large, and by all men by whom eminent attainments, modesty and true worth are revered and glorified. Dr. Marshall H. Webb, prominent in his profession, stricken in the prime of life and in the ripeness of his intellectual powers. Vain were it here to name his many virtues, or linger amid the details of a career, short but brilliant, and so well known to us all. Indeed I feel my humble abilities inadequate to accord the meed of justice his memory deserves, though my heart proclaims his worth, and our great loss in a language more deep and more eloquent than words. His busy mind and willing hands are now stilled in "death's cold embrace; the sunshine of his marked personality and his genial ways to those who knew him is forever extinguished; no longer will the accents of his voice, ever freighted with wisdom and goodly advice, be heard at our council board;" and as we instinctively turn to greet the once familiar form, absence only too pathetically and too truly reminds us that indeed of him there are

"Deposited upon the silent shore
Of memory, images and precious thoughts
That shall not die and cannot be destroyed."

Our late President, the honored and lamented William H. Goddard, like a noble tree which has withstood the blast of many winters, at last has succumbed to the sturdy and relentless strokes of time and now lies prone with all its glorious fruits full upon it. What happiness, what consolation must fill our breasts upon the reflection that this association, while it honored him with the highest distinction within its bestowal, and to which his faithful services for so many years as its Treasurer so justly entitled him, it at the same time conferred upon him a boon which may be honorably craved by any member, and for the enjoyment of which he felt with a prophetic impulse that this would be his last opportunity. Our science has suffered a great loss indeed. True, brighter names may adorn her page, achievements greater than his may shed more lustre upon her; but dentistry ne'er nurtured a son more true to her principles, more zealous for her advancement, more regardful of her integrity, more worthy of her traditions or more deserving of her honors. Gentlemen, membership in your association was a voluntary act on my part. To cast my feeble abilities with the standard-bearers of our specialty was a duty I felt I owed to the profession of my choice. Imbued with these sentiments I can scarcely realize, although ambition is laudable, that I should so soon be called upon to preside over a body of men, to whose talents and abilities and labors for the cause in the last twenty-three years is mainly due the present status of dentistry, a profession now recognized as a science and an art, not alone in the United States of America, but in every enlightened nation of the world.

Some here present may question my assertion as to whether this association is entitled to the proud distinction for which I have just given it credit, as there were other organizations in the field, not to mention the good work accomplished by our dental colleges in providing a higher grade of education. But what other organization required the adoption of a code of ethics and the degree of doctor of dental surgery before delegates from local or state societies were admitted to membership? None but the American Dental Association. This very restrictive proviso in its constitution immediately placed it upon a higher plane than any of its competitors upon the principle that to secure a status as a scientific body it was necessary to invite into its fold only those who had already secured that status as individuals. She alone recognized that every fountain must be pure if the stream is to reflect from its pellucid bosom heaven's own purity. Moreover, in the transactions of this association future generations may view the progress and history of dentistry throughout the civilized world during the past twenty-three years of its existence, and were a period now to be brought to its career, so fraught with usefulness and so pregnant with grand possibilities--were not another page to be added to its vast contributions to our well beloved science, the record of what it has already accomplished, and the important role it has played in the arena of dental thought would constitute a monument worthy the hands that reared it, and which the remorseless tooth of time can ne'er efface. Every association, in fact every individual, has a mission to perform. Our special mission is the alleviation of human suffering. What greater incentive beyond the sordid gain of filthy lucre to stimulate the power within us to action? Surely it is the grandest and noblest in which the skill and talents of man can be enlisted, and is indeed Godlike in its nature. We are laborers in the field of human progress, and it is only through organized association that dental thought can be thoroughly disseminated and usefully applied. The advantages of personal interchange of thought evolved by discussions which arise in our assemblies are not reached by merely reading the journals in the seclusion of our offices—there is something in this friction of mind which stimulates to action, engenders a high-toned emulation, practically induces investigation, and leads to higher development. Much has been accomplished, yet we are only at the threshold of our science. Through our labors many new channels for thought and research have been opened, and if the flood sometimes has seemed to spread too far and lose itself in shallow or sandy places, it has nevertheless tended to fertilize them in the end. Then, therefore, with the animating knowledge of what our society has been, what it now is, and what our hopes desire it may be, let our watchword be "Onward and upward." And as the great chieftains and mighty intellects of ancient Rome, by their abilities in the forum and their prowess upon the fields of war, bequeathed to their fellow citizens their highest and proudest distinction--*a citizen of Rome*--so, upon the peaceful plains of science, and within the walks of our own specialty, with every energy and every heart enlisted under the banners, may similar honors be earned and distinguish "a member of the American Dental Association."

